# Navigating the challenges of lipid nanoparticle formulation: the role of unpegylated lipid surfactants in enhancing drug loading and stability†

**DOI:** 10.1039/d3na00484h

**Published:** 2023-12-18

**Authors:** Cameron Hogarth, Keith Arnold, Steve Wright, Heba Elkateb, Steve Rannard, Tom O. McDonald

**Affiliations:** a Department of Chemistry, University of Liverpool Crown Street Liverpool L69 7ZD UK; b Material Innovation Factory, University of Liverpool Liverpool L7 3NY UK; c Department of Materials, The University of Manchester Oxford Road Manchester M13 9PL UK Thomas.mcdonald@manchester.ac.uk; d Henry Royce Institute, The University of Manchester Oxford Road Manchester UK

## Abstract

Lipid nanoparticles have proved an attractive approach for drug delivery; however, the challenges of optimising formulation stability and increasing drug loading have limited progression. In this work, we investigate the role of unpegylated lipid surfactants (helper lipids) in nanoparticle formation and the effect of blending helper lipids with pegylated lipid surfactants on the formation and stability of lipid-based nanoparticles by nanoprecipitation. Furthermore, blends of unpegylated/pegylated lipid surfactants were examined for ability to accommodate higher drug loading formulations by means of a higher weight percentage (wt%) of drug relative to total mass of formulation components (*i.e.* drug, surfactants and lipids). Characterisation included evaluation of particle diameter, size distribution, drug loading and nanoformulation stability. Our findings demonstrate that the addition of unpegylated lipid surfactant (Lipoid S100) to pegylated lipid surfactant (Brij S20) enhances stability, particularly at higher weight percentages of the core material. This blending approach enables drug loading capacities exceeding 10% in the lipid nanoparticles. Notably, Lipoid S100 exhibited nucleating properties that aided in the formation and stabilisation of the nanoparticles. Furthermore, we examined the incorporation of a model drug into the lipid nanoparticle formulations. Blending the model drug with the core material disrupted the crystallinity of the core, offering additional potential benefits in terms of drug release and stability. This comprehensive investigation provides valuable insights into the interplay between surfactant properties, core material composition, and nanoparticle behaviour. The study enhances our understanding of lipid materials and offers guidance for the design and optimisation of lipid nanoparticle formulations.

## Abstract 2

The study emphasises non-pegylated helper lipids functioning as both surfactants and contributors to the lipid nanoparticle core, enhancing stability at higher core percentages and potentially disrupting core crystallinity in blended formulations.

## Graphical abstract

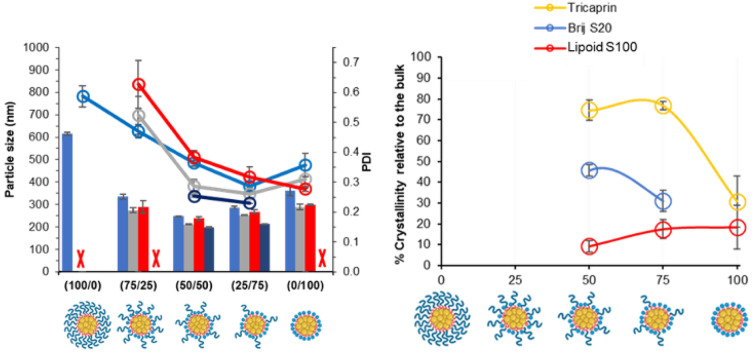

## Introduction

The widespread use of mRNA lipid nanoparticle-based vaccines employed worldwide during the COVID-19 pandemic has demonstrated the value of lipid nanoparticles (LNPs) as a carrier system. As a result, there is increased interest in using LNPs to enhance the treatment of various other conditions and diseases such as malaria to human immunodeficiency virus.^1,2^ However, there is still much that can be done to advance and understand this technology. LNPs are typically prepared by a precipitation method with water as the antisolvent. This can be achieved by microfluidics^3^ and flash nanoprecipitation. In the method of flash nanoprecipitation, the drug and or lipid is dissolved into an organic phase which is then injected into an aqueous medium which induces lipid and/or drug supersaturation followed by nucleation. Nuclei form and then grow until the concentration of material dips below that of the critical nucleation threshold whereby only growth occurs.^4^ In order to obtain greater control over formulations and achieve a lower average nanoparticle size and distribution the growth phase must be limited. Growth of nanoparticles can be distinguished by three methods; growth by diffusion, aggregation, and Ostwald ripening.^5^ Growth by diffusion occurs as the precipitating material operates by a stepwise molecular growth of solute on the particle/nuclei surface and may be limited by increasing the degree of nucleation and increasing the formation of nuclei. Growth by aggregation occurs where two or more nuclei come together to form a larger aggregate, such aggregation events may be limited by the use of surfactants to enable the production small and monodisperse nanoparticles.^5^ Ostwald ripening is a process by which larger particles grow at the expense of smaller ones, driven by the preferential dissolution and redeposition of material. It has been reported that Ostwald ripening may be limited/prevented by selecting a core material with a log *P* value at approximately 12 or above.^6–8^ The ability for a surfactant to stabilise a colloids depends heavily on the properties of the surfactant.^9^ There are various categories regarding surfactant charge for example anionic, cationic, zwitterionic and non-ionic surfactants. Non-ionic surfactants may be used to stabilise dispersions by employing steric repulsion, meanwhile ionic surfactants stabilise dispersions by means of electrostatic stabilisation and/or electrosteric.^10^ In some cases large-screening approaches using various types, combinations and amounts of surfactant have been used,^11–13^ however such an approach can be time consuming and inefficient. Attempts to streamline this process have involved identifying the design rules for nanoparticle production, one way this has been done is by characterising surfactants by their various properties for example the hydrophilic–lipophilic balance (HLB) scale. The hydrophilic–lipophilic balance (HLB) scale categorises non-ionic surfactants based on their ratio of molecular weight of hydrophilic and hydrophobic components. The HLB scale can aid in surfactant selection to result in good performance, and defines various HLB ranges for different uses.^14^ Ionic surfactants such as cationic, anionic and zwitterionic cannot be assigned a true HLB as the weight percentage calculation is skewed by presence of charge, which enhances the hydrophilic component of the surfactant making it more hydrophilic. Approximate values for HLB may be determined experimentally by the ionic surfactant's solubility or dispersibility in water; no dispersibility in water HLB 1–4, poor dispersion HLB 3–6, milky dispersion after vigorous agitation HLB 6–8, stable milky dispersion HLB 8–10, translucent to clear dispersion HLB 10–13 and a clear solution HLB 13+.^14^ Typically for lipid nanoparticle formulations surfactants have been categorised into two distinct types; pegylated lipids and unpegylated lipids.^15^ Pegylated lipids are typically a lipid which has been conjugated to a polyethylene glycol chain, which can enhance circulation times of nanoparticles *in vivo* and provide colloidal stability with the aid of a steric barrier.^16^ Meanwhile, unpegylated lipids are typically ionically neutral *i.e.* zwitterionic phospholipids such as dioleoylphosphatidylethanolamine (DOPE) and therefore offer favourable biocompatibility.^17,18^ Lipoid S100 is derived from soy lecithin containing 95% phosphatidylcholine and as such is an unpegylated lipid. Lecithin compounds are naturally found in biological membranes and Lipoid S100 when formulated as nanoparticles has been shown not to exhibit any increases in cytotoxicity when compared to other triglycerides or plant oils.^19–21^

In general, unpegylated lipids that have been demonstrated to produce relatively stable formulations below 25 °C,^22^ consequently various lipid-based nanoparticles stabilised by similar phospholipids have been marketed.^23^ Phospholipids are often termed ‘helper lipids’ due to their role in enhancing delivery efficiency and aiding nanoparticle formation.^16,24^ Additionally, the use of specific lipids have been shown to alter the biological behaviour of lipid nanoparticles, for example, increasing antigen expression,^25^ or accumulation of nanoparticles within the lymph nodes.^26^ However, there is little information in literature on the mechanism for their function in aiding nanoparticle formation. This knowledge gap can make the production of new lipid nanoparticle formulations more time consuming. Furthermore, it is widely reported that LNP formulations typically suffer from low drug loading typically <10 weight% (wt%) based on the percentage of the mass of drug as a percentage of the total formulation (including the surfactants, lipids and drug/payload).^27,28^ For many formulations, this low loading can be attributed to being dominated by a large wt% of surfactant relative to the core material. Understanding how different types of surfactants influence the formation/stability of LNPs and increasing drug loading would benefit the field.

**Fig. 1:**
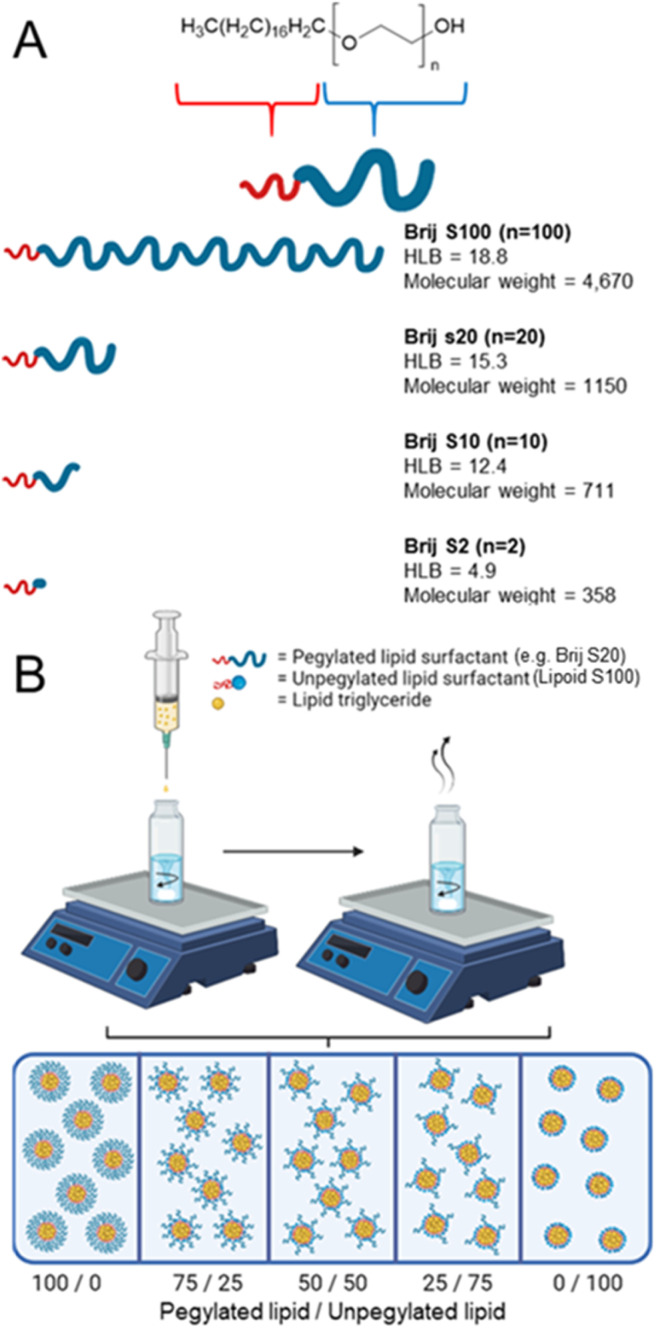
Investigating the role of surfactant and lipid type on the properties of the nanoparticles formed (A) depicts a series of linear pegylated lipids to be formulated to investigate any trend in surfactant properties. Each surfactant is composed of the same stearyl hydrophobic component, while differ in the chain length of the hydrophilic polyethylene glycol block. (B) Schematic overview of a strategy to blend both pegylated and unpegylated surfactants to assess nanoparticle formation control and stability at increased wt% of lipid triglyceride when formulating solid lipid nanoparticles.

In this work, we investigate how unpegylated lipids aid nanoparticle formation compared to pegylated lipids. We employed a series of pegylated lipid surfactants with varying hydrophilic chain lengths, conducting a systematic investigation into how surfactant properties, such as HLB influence the formation and stability of lipid nanoparticles through flash nanoprecipitation, as illustrated in Fig. 1A. Furthermore, we will also make direct comparisons between the pegylated lipid surfactant and the unpegylated lipid surfactant before examining how blends of both unpegylated and pegylated lipid surfactants influence the formation of stable formulations with a higher wt% of core material thus increasing potential drug loading, Fig. 1B. Finally, to investigate the drug loading potential, a model drug is blended into the most promising formulations demonstrating that high drug loading formulations can be achieved.

## Results and discussion

### Effect of HLB on particle formation and stability

The four linear pegylated lipid surfactants were used to formulate the solid lipid tricaprin at 14 wt%. To better understand the relationship between the HLB of the surfactant and the properties of the resultant lipid nanoparticles, Brij surfactants were selected. These surfactants were composed of the same stearyl chain for the hydrophobic components meanwhile varying the chain length of the hydrophilic polyethylene glycol (PEG) from 2, 10, 20 and 100 units. The number of PEG repeat units is shown in the name of the surfactant, *i.e.* Brij S10 contains 10 PEG repeat units. Information on the properties of the surfactants is shown in Fig. 1A. Tricaprin was nanoprecipitated into a fixed volume of each of the Brij surfactants Brij S20 and Brij S100. Due to aqueous solubility limits of the surfactants with shorter PEG chains, the Brij S2 and Brij S10 were instead dissolved in the organic phase along with the triglyceride tricaprin.

**Fig. 2:**
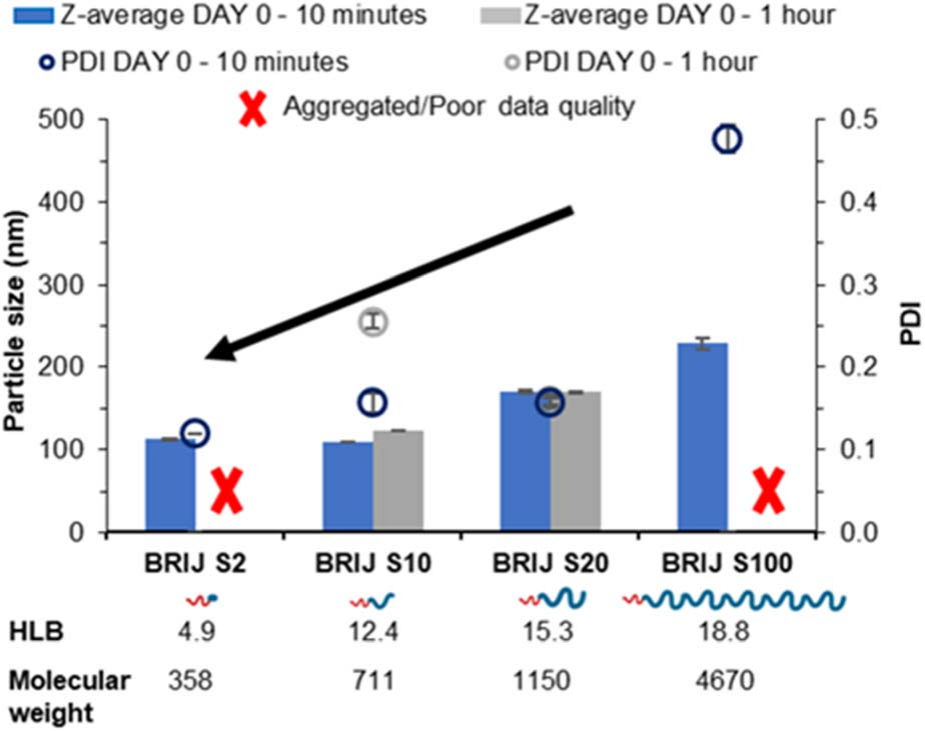
Data collected measuring both particle size and polydispersity of a series of tricaprin nanoparticle formulations stabilised by various Brij surfactants at equal mol%.

Upon nanoprecipitation, the formulations were analysed by dynamic light scattering (DLS) to measure the size distribution of any particles formed. The mean diameter and the polydispersity index (PDI) were measured 10 minutes post formulation (Fig. 2) and showed that both the diameter and PDI decrease with decreasing PEG chain length of the pegylated lipid surfactants. After an hour, both samples stabilised by the Brij surfactants with the shortest or longest PEG chains were no longer suitable for DLS measurements; the formulation stabilised by Brij S2 possessed a shimmering effect, indicating the presence of anisotropic crystal formation (Fig. S1A†) likely caused by insufficient steric stabilisation provided by the surfactant thus resulting in aggregation. Meanwhile, the formulation stabilised by the longest PEG chain (Brij S100) contained aggregates (Fig. S1C†), potentially caused by depletion flocculation due to the presence of excess polymer.^29^ The formulation with a PEG chain length of 10 repeat units (Brij S10, HLB of 12.4) the nanoparticles were found to increase in diameter within 1 hour of production. This suggests that the chain length of PEG was too short to provide sufficient steric repulsion to stabilise the growing nuclei. Meanwhile, the formulation of Brij S20 showed no increase in diameter and contained no visible particles under the optical microscope (Fig. S1B†), indicating that the particles had sufficient steric stabilisation. Therefore, Brij S20 was identified as the most efficient surfactant for further investigation. In combination, this data suggested that at the surfactant with lower HLB values (thus lower solubility in the aqueous phase) might share a ‘nucleation like’ behaviour similar to that of the triglyceride, forming more nucleation sites which may in turn provide a decrease in initial nanoparticle size and PDI. However, without a sufficiently long PEG chain the surfactant was not able to provide an adequate steric barrier to give longer term stability.

### Nucleation behaviour of non-pegylated ‘helper’ lipids surfactants

**Fig. 3:**
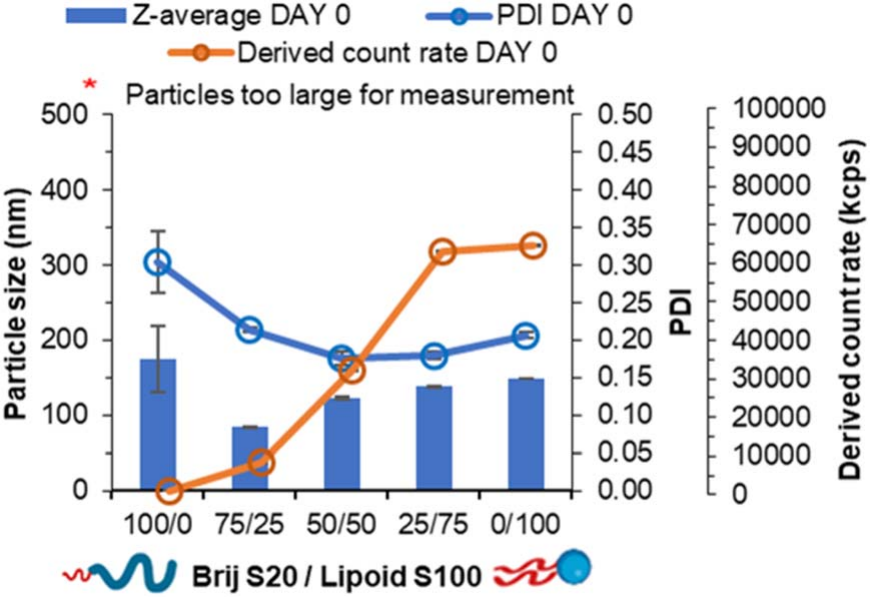
Data collected both particle size, polydispersity and derived count rate of a series of pegylated/unpegylated lipid surfactant blends with no triglyceride.

Nucleation of material has previously been attributed to the degree of supersaturation with greater supersaturation resulting in greater nucleation of material.^30^ Furthermore, log *P* has been shown as an indicator of nucleation with higher log *P* resulting in a higher degree of nucleation of material and smaller and more uniform nanoparticle formulations.^7^ We hypothesised that helper lipids may aid nanoparticle formation as they would nucleate alongside the other core components in a similar manner to Brij S2 due to sharing similar log *P*, (Brij S2; *C* log *P* = 6.5, Lipoid S100; *C* log *P* ∼4.6, note Lipoid S100 is a natural blend of various alkyl chain length and saturation of phosphatidylcholine while is predominantly of linoleic acid at 65%). To test this hypothesis, formulations were prepared in the absence of core material *i.e.* no tricaprin; therefore, the only components would be the surfactants Brij S20 and Lipoid S100. The two surfactants were prepared at equal mass ratios 100/0, 75/25, 50/50, 25/75 and 0/100 and analysed by DLS to obtain particle size information and the derived count rate (a measure of the scattering intensity). At 100% Brij S20, objects with a mean diameter of 174 nm, a PDI of 0.3 and a derived count rate of 488 kilo counts per second. The weak scattering and polydispersity of the sample provided evidence of the it being composed of micelles. On the other hand, as Lipoid S100 was introduced into the formulation, the derived count rate increased as nanoparticles were likely formed (Fig. 3), and at 100% Lipoids S100 the highest derived count rate was detected. Additionally, with increasing Lipoids S100 content the mean diameter also increased likely as the mass of material in the core of the nanoparticles was increased. From this data it was plausible to assume that upon injection, Lipoid S100 nucleates and subsequently grows to form nanoparticles.

### Comparison of lipid nanoparticle size and stability using either pegylated or unpegylated surfactants

To investigate how the unpegylated lipid surfactant Lipoid S100 influenced nanoparticle formation, a direct comparison was made between the unpegylated lipid, Lipoid S100 and the pegylated lipid Brij S20. Both surfactants were used at an equal mol% to stabilise triglycerides of increasing log *P*; tricaprin (*C* log *P*, 13.1), trimyristin (*C* log *P*, 19.5) and tristearin (*C* log *P*, 25.8). Increasing the log *P* of the core lipid would result in an increase the degree of supersaturation and therefore increase the degree of nucleation.^7^ Such an increase in the number of nuclei could lead to more aggregation events if an inefficient or insufficient surfactant was used.^5^ As a result, a comparison can be made of how the properties of the different surfactants may enable the production of a stable formulation.

**Fig. 4:**
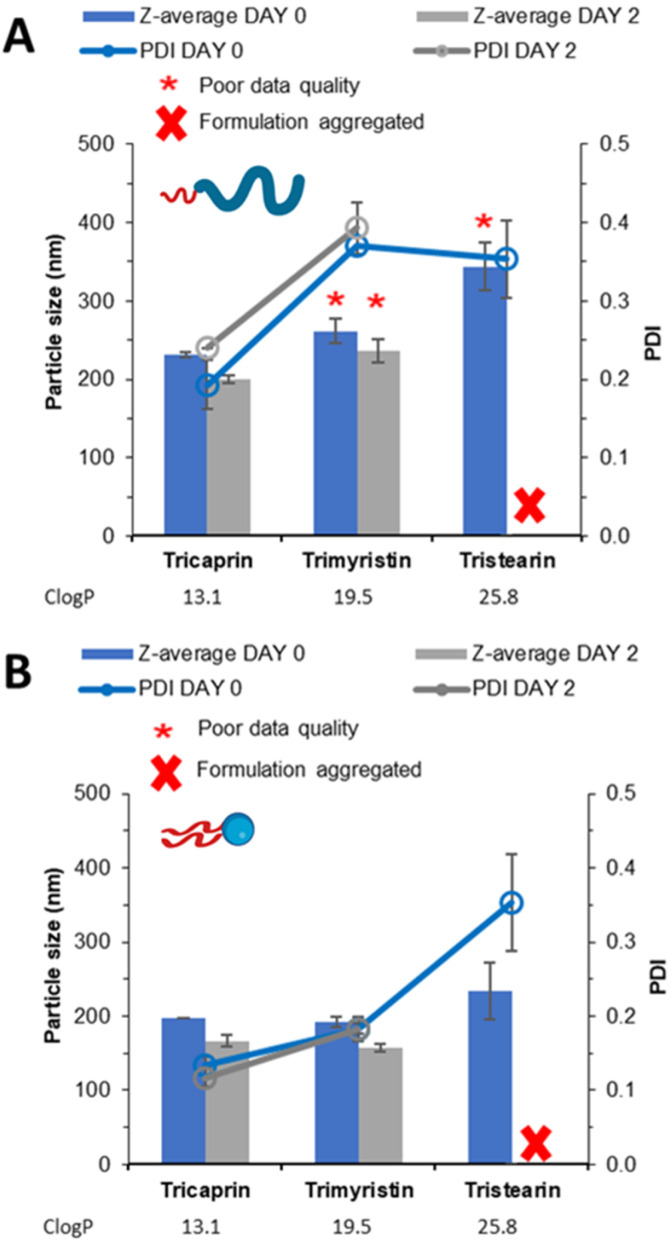
Data collected measuring both particle size and size distribution of a series of triglyceride formulations stabilised on an equal mol% by (A) pegylated lipid surfactant (Brij S20) (B) unpegylated lipid surfactant (Lipoid S100).

The samples were analysed by DLS (Fig. 4A) showing that increasing log *P* from tricaprin to tristearin while using the pegylated lipid surfactant Brij S20 resulted in poor DLS data quality (likely due to the presence of large aggregates). However, when the different lipids were formulated using Lipoid S100 (Fig. 4B) an increase in particle size and polydispersity with increasing log *P* was observed. It is clear from Fig. S2A† that samples of trimyrisitin stabilised by Brij S20 contain aggregates due to the presence of a shoulder on the correlation curve, meanwhile when Lipoid S100 was used as a stabiliser the correlation curve was smooth and consistent, Fig. S2B.† All samples were analysed on day 2 once the organic solvent had evaporated to assess short term stability. On day 2, the instability of the trimyristin and tristearin samples stabilised by Brij S20 had become more apparent due to noticeable sedimentation and precipitation. Meanwhile, trimyristin stabilised by unpegylated lipid (Lipoid S100) remained stable. Samples of both pegylated and unpegylated lipid stabilising tristearin contained visible aggregates and were therefore unsuitable for DLS measurement. Hence all four of these samples were characterised by optical microscopy Fig. S3,† showing the presence of aggregates for each of the samples Brij S20/trimyristin (Fig. S3A†), Brij S20/tristearin (Fig. S3C†) and Lipoid S100/tristearin (Fig. S3D†), while shows no sign of visible aggregates for the formulation of trimyristin stabilised by Lipoid S100 (Fig. S3B†) This data set suggests that Lipoid S100 results in greater stabilisation, which may be due to faster stabilisation of growing nuclei by limiting the number of aggregation events in comparison to pegylated lipids.

Given the greater efficacy of the unpegylated lipid surfactant (Lipoid S100) in particle stabilisation, compared to the pegylated lipid surfactant Brij S20, we also investigated the potential to increase the content of the lipid (tricaprin) in the formulation. This would be attractive for a nanoformulation as the higher mass of lipid core in the formulation potentially offers the opportunity for also high drug loadings. We found that using Lipoid S100 as the surfactant enabled stable formulations with 50% mass of lipid to be produced, while the pegylated surfactant was only able deliver stable formulations at 25% mass (Fig. S4†). Overall, this data set further suggests that unpegylated lipid surfactants provide more stable nanoformulations compared to the pegylated lipid. This behaviour may be due to the nucleation of the Lipoid S100 alongside the triglyceride tricaprin which may then limit the number of aggregation events.

### Investigation of pegylated/unpegylated lipid surfactant blends

Blending surfactants is a strategy that may be employed to formulate nanoparticles that offer the benefits of both the pegylated and unpegylated lipid surfactants. This way, a blend may offer good control over the nanoparticle formation while maintaining steric stability enabling prolonged circulation time.^24^ It was hypothesised that by employing the blend of lipid surfactants (pegylated and unpegylated) it may be possible to develop formulations of a higher wt% of core material. To investigate the effect of blending surfactants, five formulations were prepared from 100% pegylated surfactant to 100% unpegylated surfactant with blends of surfactants produced at 25% intervals and the loading of tricaprin which was increased from 14 to 25, 33 and 40 wt%.

**Fig. 5:**
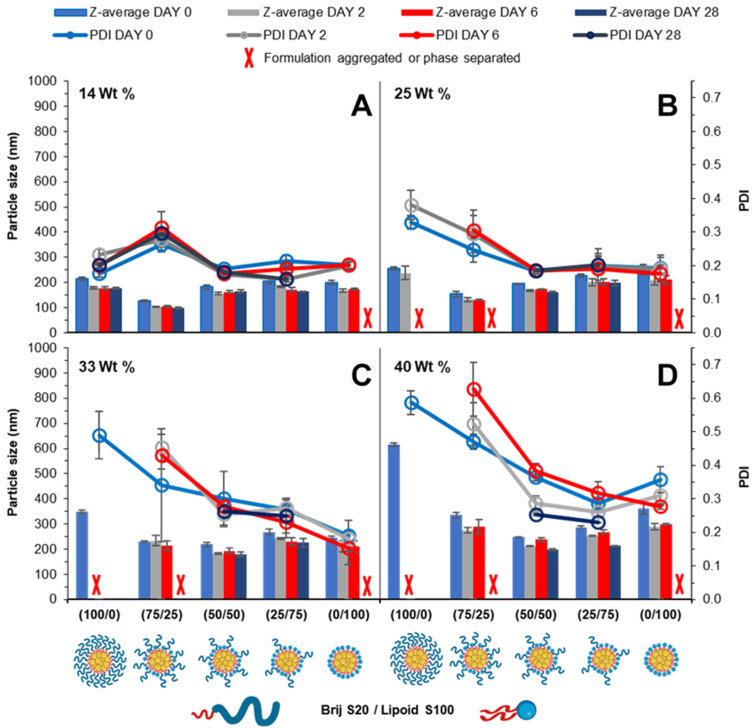
Particle size and size distribution obtained by DLS over a 28 day period. Formulations varied in pegylated/unpegylated lipid surfactant blends on a mass ratio. Data also examines the effect of increasing wt% of tricaprin 14 (A), 25 (B), 33 (C) and 40 (D) wt%. Samples were prepared in triplicate and error bars calculated on standard deviation between sample measurements. Samples were stored at 22 °C in deionised water.

Immediately after injection obvious differences in the amount of light scattering were noticeable as change in turbidity of the samples (Fig. S5†). Generally, the surfactant composition had a large influence on formulation turbidity at 14 wt%, and each formulation increased in turbidity as the wt% increased and each blend became indistinguishable, likely a consequence of increased particle size and/or concentration of nanoparticles. DLS was also used to measure the mean particle diameter and PDI over a 28 day period. At 14 wt% tricaprin the inclusion of 25% Lipoid S100 resulted in the nanoparticle formulation with the smallest size, which explained why this sample had the lowest turbidity (smaller particles have much weaker light scattering). When the amount of Lipoid S100 was increased in the formulation there was a slight increase in nanoparticle size. This was attributed to Lipoid S100 potentially including itself within the core of nanoparticles at 14 wt%. Generally, the particle diameter and PDI decreased with increasing unpegylated lipid surfactant within the surfactant blend. Additionally, there was generally an increase in particle size and polydispersity with increasing wt% of tricaprin (Fig. 5). Over the prolonged period of 28 days the formulations at 0/100 (Brij S20/Lipoid S100) were found to be unstable due to the occurrence of phase separation, poor long-term stability was also seen for 100/0 and 75/25 (Brij S20/Lipoid S100) at lipid loadings of ≥25 wt%, as observed as visible non-spherical particles (Fig. S6†). Zeta potential measurements on the samples revealed a charge less than +10 mV for each surfactant blend, suggesting the formulations were stabilised solely by steric stabilisation, Fig. S7.† These experiments showed the benefit of the use of blends of surfactants; formulations containing a high proportion of unpegylated lipid such as 50/50 or 25/75 unpegylated were able to maintain stability likely a result of the steric stability provided by the Brij S20.

Cryo-SEM was also employed to visualise the particles of the tricaprin nanoparticles at 14 wt% stabilised by 50/50 (Brij S20/Lipoid S100) Fig. S8A.† Measurement of the nanoparticles from the cryo-SEM image revealed an average particle diameter of 131 nm Fig. S8B,† in agreement with data obtained by DLS (157 nm) after considering the effects of solvation sphere that is included in the DLS measurement. The cryo-SEM analysis also showed that the sample was composed a range of particle sizes from ∼40 nm up to ∼400 nm. This breadth in the size distribution might have been due to differences in the nucleation behaviour of the tricaprin and lipoid S100 resulting in differences in the duration over which the particles could grow.

**Fig. 6:**
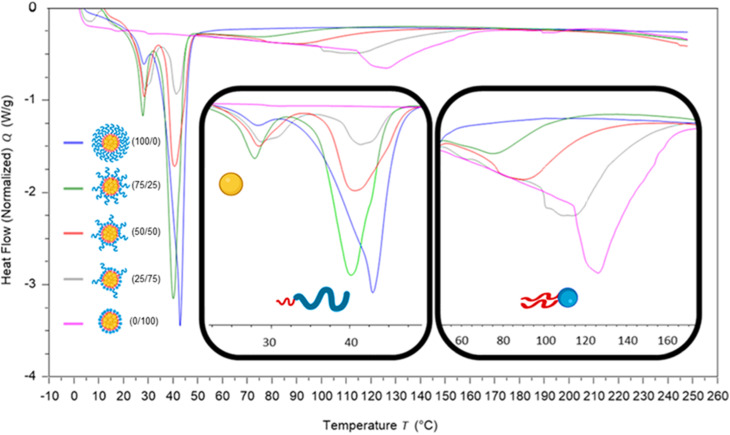
Overlay of DSC traces for each of the nanoparticle formulations at 14 wt%. The two inserts show the regions 22.5–50 °C; indicating the effect of surfactant composition on the crystallinity of the core (tricaprin) and pegylated lipid surfactant, and 50–170 °C; indicating the effect of surfactant composition on the crystallinity of Lipoid S100.

**Fig. 7:**
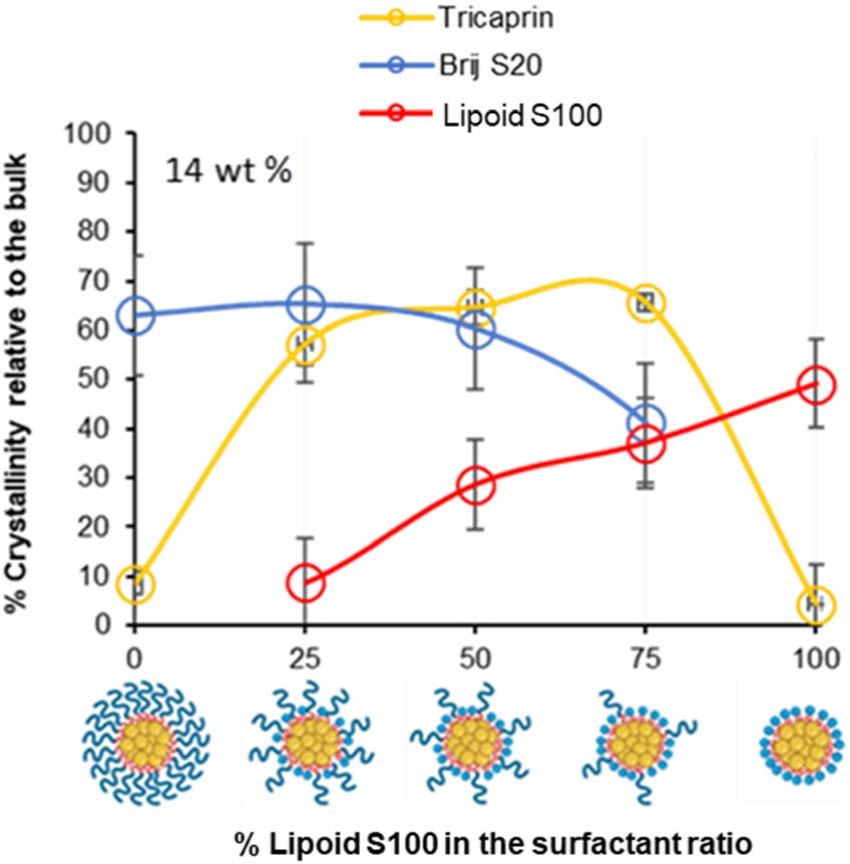
Graph showing the relationship between surfactant composition and crystallinity of tricaprin within the core at 14 wt% as determined by DSC analysis. Also informs of surfactant crystallinity.

To investigate how the surfactants influenced the crystallinity of the nanoparticles the thermal properties of the various individual materials were analysed by differential scanning calorimetry (DSC). Analysis of the individual components showed that tricaprin had a melting point of ∼32 °C, Brij S20 showed a broad melting point of ∼46 °C and Lipoid S100 ∼160 °C (Fig. S9†). The 14 wt% lipid formulations with varying surfactant compositions were then analysed by DSC. Fig. 6 displays an overlay of the thermograms for each of the nanoparticle formulations and how the melting transitions changed according to the surfactant composition. The endotherms occurring at approximately 28 °C correspond to tricaprin and thus the core crystallinity. Interestingly, the core appeared virtually amorphous at a surfactant composition of 0/100 (Brij S20/Lipoid S100), meanwhile is most intense when a blend of 75/25 Brij S20 and Lipoid S100 was used. As expected, the Lipoid S100 endotherm region peak intensity broadened and became less intense as the percentage of Lipoid S100 is decreased, meanwhile the endotherm for the alternative Brij S20 became sharper and increased in intensity. Smaller and broader melting endotherms suggest a reduction in crystallinity likely due to disruption of one surfactant by another. Fig. 7 displays how the crystallinity of tricaprin at 14 wt% is reduced relative to bulk tricaprin when nanoparticles are produced using either surfactant Brij S20 or Lipoid S100.

This reduction in crystallinity was likely the consequence of disruption of material crystallinity due to nanoformulation compared to a bulk material.^31^ Furthermore, it has been reported that surfactant compositions may influence the control of nanoparticle crystallinity.^31^ Lipoid S100 appeared to disrupt the crystallinity of tricaprin more than Brij S20. This may be a consequence of the two alkyl chains of the phospholipid causing greater disruption of the tricaprin within the core rather than the single alkyl chain of Brij S20. Alternatively, as Lipoid S100 has been proven to form nanoparticles itself some Lipoid S100 may have blended within the core alongside tricaprin resulting in greater disruption, thus explaining the changes in the crystallinity in the different components of the formulations is shown in Fig. 7. Interestingly, as the two surfactants were blended the overall crystallinity of the tricaprin increased dramatically, Bunjes *et al.* have previously suggested upon establishing a uniform surfactant layer, interactions between the surfactants are strong and the fluidity of the membrane layer is decreased. As a result, the crystalline tendencies of the core such as polymorphic transitions are reduced.^32^ Therefore, it is possible that by blending the pegylated surfactant with the unpegylated lipid surfactant the phospholipid membrane is disrupted resulting in increased fluidity, and an overall increase in crystallinity due to less interactions between the hydrophobic tails of the surfactants and the core lipid. Nevertheless, further investigation would be required to understand the precise cause of this difference.

**Fig. 8:**
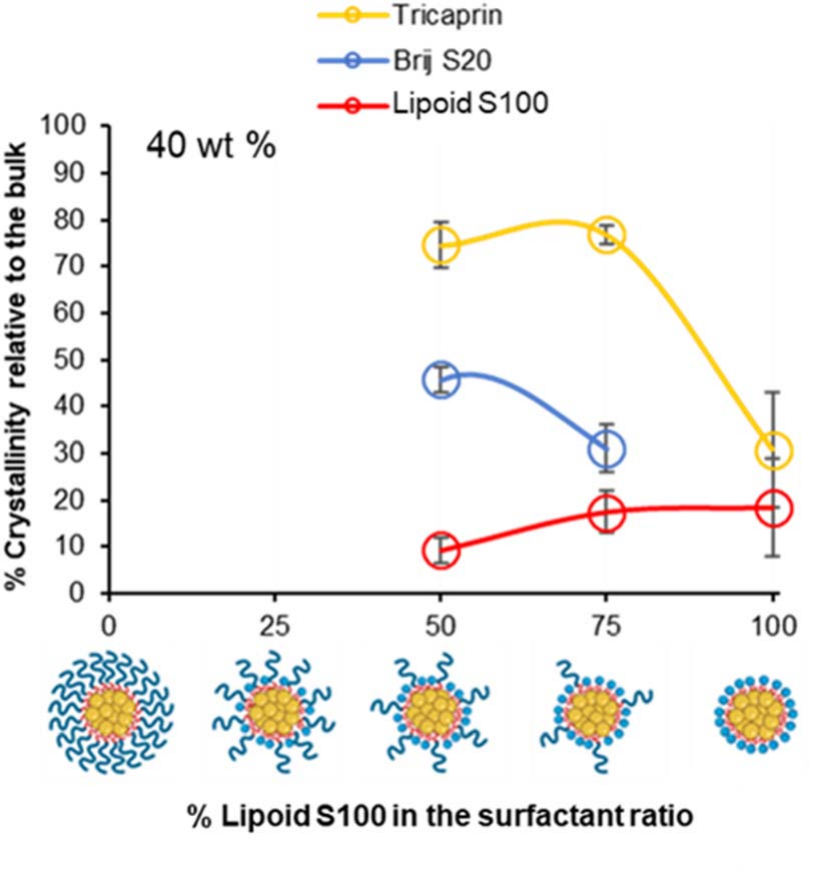
The relationship between surfactant composition and relative crystallinity of tricaprin within the core at 40 wt% as determined by DSC analysis.

To investigate if the crystallinity of the components changed with at increase in the lipid content in the formulation, DSC analysis was also performed on various formulations at higher wt% of 40% tricaprin (Fig. 8). Only formulations containing at least 50% of Lipoid S100 are shown as the formulations with lower amounts of Lipoid S100 did not display long term stability (Fig. 5D). For the formulations tested, an increase in enthalpy of tricaprin melting was observed compared to 14 wt%, meanwhile the enthalpy of surfactant melting decreased, and peaks underwent broadening. The peak for Lipoid S100 also appeared to shift to a lower melting temperature. Overall, these changes suggested an increase in core crystallinity with increasing wt% from 14 wt% to 40 wt%. The thermograms are shown in Fig. S10,† and reveal that at 40 wt% tricaprin was found to possess two peaks when stabilised by 100% Lipoid S100 with the first melting peak at ∼17 °C and the second at ∼30 °C. Thus, suggesting the presence of two distinctly different crystal forms of tricaprin with the earlier being liquid at room temperature and the latter being solid. Therefore, the overall structure of the nanoparticle would adopt a nanostructured lipid nanocarrier structure rather than a solid lipid nanoparticle.^33,34^

Overall, as the wt% of core material (tricaprin) was increased to 40 wt%, the crystallinity of surfactants decreased while the crystallinity of tricaprin increased. For example, at 50/50 (BrijS20/Lipoid S100) there was an average increase in component crystallinity relative to bulk material for tricaprin. Meanwhile, Brij S20 decreased on average by 14.5% from 60.3% to 45.8% and Lipoid S100 decreased by 19.3% from 28.5% to 9.2%. The increase in core crystallinity of tricaprin was likely caused by a decrease in the overall disruption caused by the alkyl chains entering the core of the nanoparticle as the size of the core and/or number of nanoparticles is increased.

### Incorporation of a model drug into pegylated/unpegylated lipid surfactant blends

**Fig. 9:**
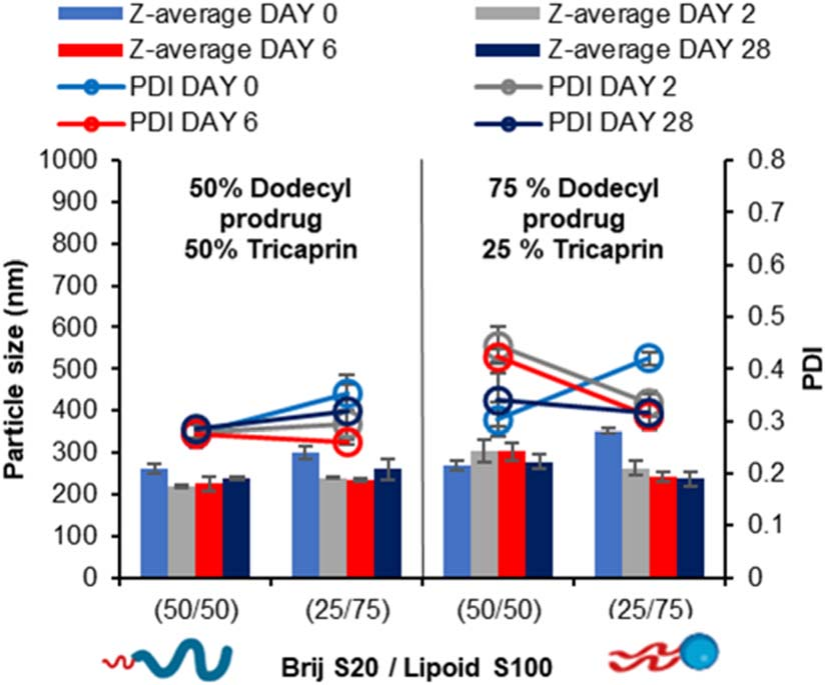
Particle size and size distribution obtained by DLS over a 28 day period. Formulations at 40 wt% core yet varied in core composition at both 50% and 75% dodecyl prodrug loading while also varied in surfactant composition 50% Brij S20/50% Lipoid S100 and 75% Brij S20/25% Lipoid S100. Samples were prepared at in triplicate and error bars calculated on standard deviation between sample measurements. Samples were stored at 22 °C in deionised water.

In our prior work, we demonstrated that the overall increase in core crystallinity may be overcome by the introduction of drug/prodrug into the core.^7^ Given the stability of the 40 wt% lipid formulations with the surfactant ratios of 50/50 and 25/75 BrijS20/Lipoid S100 surfactant blends, these samples were identified as best candidates for formulating with a model drug. A dodecyl prodrug was identified in our previously work, as this compound formed some of the most stable nanoparticle formulations compared to other model drug compounds.^7^ The prodrug was employed in a blend of either 50% tricaprin 50% dodecyl prodrug or 25% tricaprin 75% dodecyl prodrug, with these two components making up to 40 wt% of the complete formulation. Fig. 9 displays the mean particle size and PDI values obtained by DLS analysis over a 28 day period. Each blend produced a particle formulation uniform in size and PDI with average particle size ranging between ∼200 and 300 nm. The corresponding size distribution graphs and correlation curves are also displayed by Fig. S11.† Zeta potential measurements revealed mean particle charge of −4 to 8 mV, thus suggesting the formulations were stabilised solely by steric stabilisation, Fig. S12.†

**Fig. 10:**
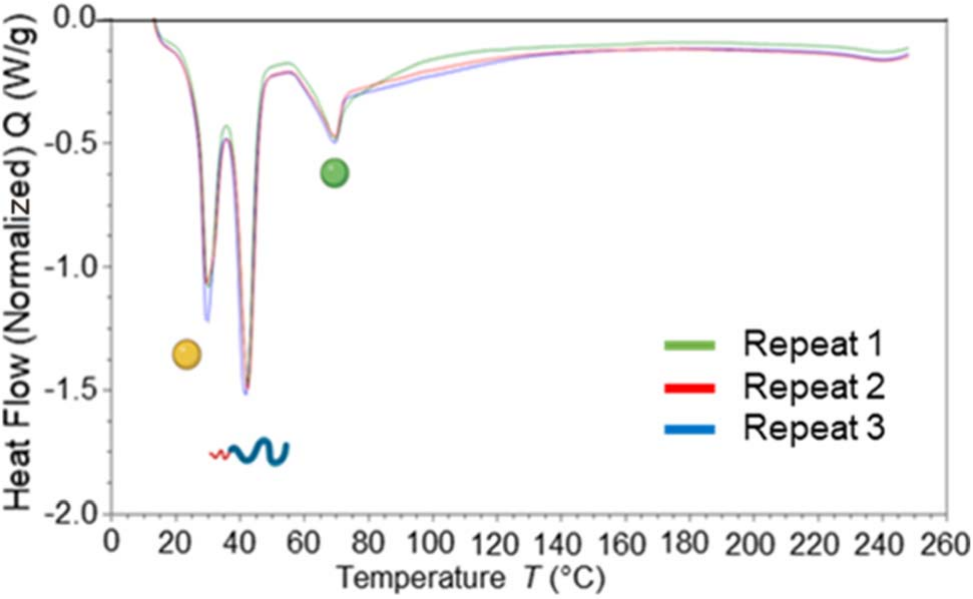
Displays an overlay of DSC traces for nanoparticle formulations prepared in triplicate with a core composition of 50% dodecyl prodrug (model drug) (green circle indicates the expected melting transition) and 50% tricaprin (yellow circle indicates the expected melting transition) while a surfactant composition of 50% Brij S20 50% Lipoid S100.

Blending of core materials has previously been shown to influence the core crystallinity,^7^ therefore DSC analysis was carried out to determine the impact of blending dodecyl prodrug alongside tricaprin. Fig. 10 displays an overlay of DSC traces for formulations at 50% dodecyl prodrug loading at a surfactant composition of 50% Brij S20/50% Lipoid S100. It was calculated the crystallinity of tricaprin reduced by ∼21% from 74.9% to 53.9% crystallinity compared to the equivalent formulation at 100% tricaprin. The single melting peak suggested that all the crystalline lipid would be solid at body temperature (unlike when triciprin was the lone material in a 40 wt% formulation). Unfortunately, it was not possible to obtain an accurate value for the crystallinity of the dodecyl prodrug due to overlapping of the dodecyl prodrug and Lipoid S100 peaks. To further investigate the crystallinity, powder X-ray diffraction (PXRD) was used to analyse the nanoparticle formulation of 50% dodecyl prodrug 50% tricaprin (surfactant composition of 50% Brij S20 50% Lipoid S100) compared to the starting materials. Due to overlapping peaks associated with the different components in the nanoparticle it was difficult to determine which material contributes to crystallinity in the formulation based on the PXRD data (Fig. S13†). Nevertheless, this study demonstrates how loading dodecyl prodrug at 50% of the core mass at a surfactant composition of 50/50 pegylated/unpegylated lipid surfactant blend resulted in disruption of the crystallinity of the core due to a decrease in tricaprin relative crystallinity.

## Conclusions

Non-pegylated surfactants such as Lipoid S100 can nucleate to form nanoparticles itself and likely helps to stabilise the growing nuclei. Blending the unpegylated lipid surfactant Lipoid S100 with pegylated lipid surfactant Brij S20 resulting in benefits such as enhanced stability at higher wt% of core material, thus enabling higher drug loading formulations. In addition, blends of prodrug and lipid at elevated core wt% have been shown to disrupt core crystallinity.

Overall, this work provides greater understanding of how the properties of the lipids within a formulation determine particle formation and stability, translating into the advantageous attributes for lipid nanoparticle formulations. These advantages include enhanced stability of highly hydrophobic materials as well as high loading (*i.e.* <10%) of drugs. Notably, the insights from this work are have the potential to be applied to materials other than lipids. Consequently, this study may be used as a guide to design and optimise nanoparticle formulations in the future; eliminating the need for a screen or reduce the scale of a surfactant screen by allowing the selection of surfactants of specific properties which may complement the material to be encapsulated. Future research should focus on the practical application of our findings in targeted organ drug delivery. Undertaking comprehensive biological studies will be pivotal in gaining a more detailed understanding of how the composition of lipid surfactants plays a crucial role in facilitating efficient endocytosis and endosomal escape, ultimately promoting active drug delivery within specific target cells.

## Experimental

### Materials

Dodecyl prodrug/drug analogue was used as synthesised by previous publication. Brij S10, Brij S20, Brij S100, tetrahydrofuran and deuterated solvents (CDCl_3_ and D_2_O) were all purchased from Sigma Aldrich and used as received apart from CDCl_3_ where 0.1% tetramethylsilane was added. Lipoid S100 was purchased from Lipoid and used as received. Tricaprin was purchased from Tokyo chemical industry and used as received. Dynasan 114 (trimyrisitn) and Dynasan 118 (tristearin) was kindly gifted from IOI Oleochemical, Hamburg. Lamivudine was purchased from Top Well Medipharma Group.

### Methods

#### General nanoparticle preparation

Method adopted for nanoparticle formulation was nanoprecipitation and was derived from a literature approach.^33^ This work, for the aqueous phase, the surfactant Brij S20 was dissolved to prepare a 1000 mL stock solution in distilled water (1 mg mL^−1^) and left overnight at 21 degrees Celsius under mechanical stirring (300 rpm). Portions of the stock solution were taken and potentially diluted further with distilled water; composition shown by Table 1. For the organic phase stock solutions of tricaprin (4 mg mL^−1^) and Lipoid S100 (24 mg mL^−1^) were prepared in tetrahydrofuran. Compositions are shown by Table 2. The organic phase was charged dropwise into the vortex of the aqueous phase contained in a 40 mL vial while mechanically stirring (800 rpm). To ensure consistency in time of injection the shot was charged by removing the plunger of a clamped syringe resulting in a steady flow through the hypodermic needle. The combined mixture was left stirring to allow evaporation of tetrahydrofuran over 2 days at a room temperature (∼22 °C) in a fume cupboard with an average air velocity of 0.35 m s^−1^. Samples were then stored at 22 °C. Note that other Brij surfactants were either dissolved in aqueous or organic phase depending on solubility.

**Table 1:** Aqueous phase composition depending on surfactant blend

Surfactant composition ratio of Brij S20/Lipoid S100	Volume Brij S20 stock solution (mL)	Volume distilled water (mL)	Total volume aqueous phase (mL)
100/0	24	0	24
75/25	18	6	24
50/50	12	12	24
25/75	6	18	24
0/100	0	24	24

**Table 2:** Organic phase composition depending on surfactant blend

Surfactant composition ratio Brij S20/Lipoid S100	Volume tricaprin stock solution (mL)	Volume Lipoid S100 stock solution (mL)	Volume neat THF (mL)	Total volume organic phase injectable shot (mL)
100/0	1	0	1	2
75/25	1	0.25	0.75	2
50/50	1	0.5	0.5	2
25/75	1	0.75	0.25	2
0/100	1	1	0	2

#### Preparation of lipid nanoparticle formulations varying in pegylated lipid Brij S20 and unpegylated lipid Lipoid S100 at elevated wt%

Formulations prepared in the same way as at 14 wt% although the concentration of the tricaprin stock solution was increased *i.e.* (25 wt%, 8 mg mL; 33 wt%, 16 mg mL; 40 wt%, 16 mg mL^−1^).

#### Preparation of lipid nanoparticle formulations varying in pegylated lipid Brij S20 and unpegylated lipid Lipoid S100 at elevated wt%

General preparation method the same although composition of organic phase adjusted. Table 3 displays an example of blends of tricaprin and dodecyl drug analogue/prodrug at 40 wt%. Stock solution concentrations; tricaprin (16 mg mL^−1^) and dodecyl drug analogue (16 mg mL^−1^).

**Table 3:** Organic phase composition depending on blends of tricaprin, dodecyl drug analogue/prodrug as well as surfactant blends

Core composition ratio tricaprin/dodecyl prodrug	Surfactant composition ratio Brij S20/Lipoid S100	Volume tricaprin stock solution (mL)	Volume dodecyl drug analogue stock solution (mL)	Volume Lipoid S100 stock solution (mL)	Volume neat THF (mL)
50/50	50/50	0.5	0.5	0.5	0.5
50/50	25/75	0.5	0.5	0.75	0.25
25/75	50/50	0.25	0.75	0.5	0.5
25/75	25/75	0.25	0.75	0.75	0.25

#### Nanoparticle characterisation methods

##### Dynamic light scattering (DLS) and zeta potential

Samples were analysed by DLS using The Malvern ZetaSizer Nano S DLS obtain a Z-average and size distribution (PDI) and zeta potential of nanoparticle dispersion. 2 mL of each sample (1.17–1.7 mg mL^−1^) was measured in standard 3 mL fluorimeter cuvettes with a pathlength of 10 mm. All measurements were carried out at 25 °C with a fixed backscattering angle of 173° using automated setting. Each sample was measured once although formulations were done in triplicate. Zeta potential was also measured using Malvern ZetaSizer Nano S. Samples were measured using automated settings and samples were measured in triplicate in a Malvern Zetasizer Nano series disposable folded capillary cell. All measurements were carried out at 25 °C.

##### Differential scanning calorimetry (DSC)

Nanoparticle formulations were dried in a glass vial by lyophilization before weighing out portions of the solid monolith into Aluminium pans. Performed by a TA DSC25. The freeze-dried nanoparticle formulations were equilibrated at 10 °C before heating to 100 °C at a rate of 10 °C per minute. The bulk samples were heated to 100 °C at a rate of 10 °C per minute before being cooled back down to 0 °C at a rate of 5 °C per minute and again heating back up to 100 °C at a rate of 10 °C per minute. Measurements were carried out in triplicate.

##### Cryogenic scanning electron microscopy (cryo-SEM)

Specimens were prepared by freezing a small volume of sample between two brass rivets, which are plunged into slushed liquid nitrogen. Rivets were transferred to a brass loading shuttle under liquid nitrogen and transferred under a nitrogen atmosphere to a preparation stage cooled to −120 °C. Anti-contaminator in the preparation stage was run at −190 °C. Fracture surfaces were created in the frozen specimen by pushing-off the upper rivet from the one held in the shuttle (using a liquid nitrogen cooled knife). The specimens were sublimed for 3 minutes oat −90 °C, to create a contrast mechanism. Fracture surfaces were coated with Pt in the preparation chamber, to make them conductive and the specimens transferred to a cooled stage in the SEM (at −160 °C, with an anti-contaminator held at −190 °C). The specimens were photographed using an in-chamber secondary electron detector using either 1.5 keV or 10 keV and a beam current of 15 pA. Mean particle size was determined by measuring ∼100 particles using by Image J.

##### Powder X-ray diffraction (PXRD)

PXRD data were collected in transmission mode on a Panalytical X'Pert PRO MPD equipped with a high throughput screening (HTS) XYZ stage, X-ray focusing mirror and PIXcel detector, using Cu Kα radiation. Data were measured on loose powder samples held on thin Mylar film in aluminium well plates, over the range 4 to 40° in approximately 0.013° steps over 60 minutes.

## Conflicts of interest

There are no conflicts to be declared.

## Supplementary Material

NA-006-D3NA00484H-s001
