# Supplementary material for: Navigating the challenges of lipid nanoparticle formulation: the role of unpegylated lipid surfactants in enhancing drug loading and stability
Source: Nanoscale Adv. 2023 Dec 18;6(2):669–79. doi: 10.1039/d3na00484h (PMC10791113; doi:10.1039/d3na00484h)
Supplement: NA-006-D3NA00484H-s001 [file NA-006-D3NA00484H-s001.pdf]

Electronic Supplementary Material (ESI) for Nanoscale Advances.
This journal is © The Royal Society of Chemistry 2023

Navigating the Challenges of Lipid Nanoparticle Formulation: The Role of Unpegylated Lipid Surfactants in Enhancing Drug Loading and Stability

Cameron Hogarth, Keith Arnold, Steve Wright, Heba Elkateb, Steve Rannard, Tom O. McDonald.

Supplementary Information

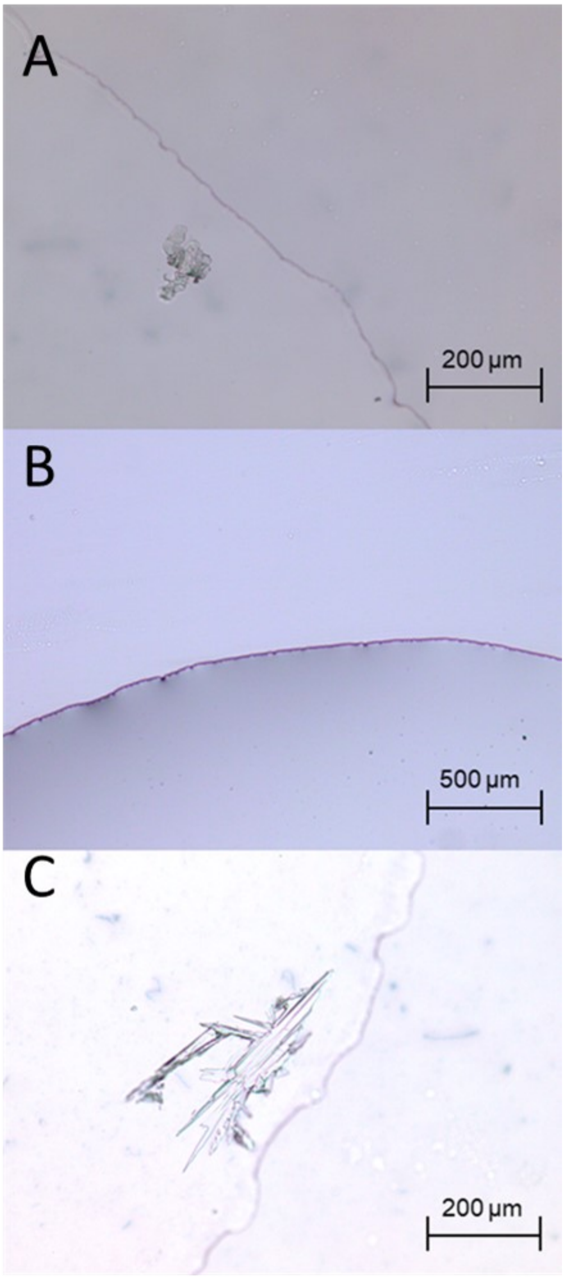

Fig. S1- Optical microscopy images of formulations stabilised by the various Brij surfactants after 1 hour. A and C highlight the instability of the formulations containing Brij S2 and S100 respectively. Meanwhile, B shows no sign of visible aggregates when stabilised by Brij S20. Note that the magnification used for B is different to A and C, this

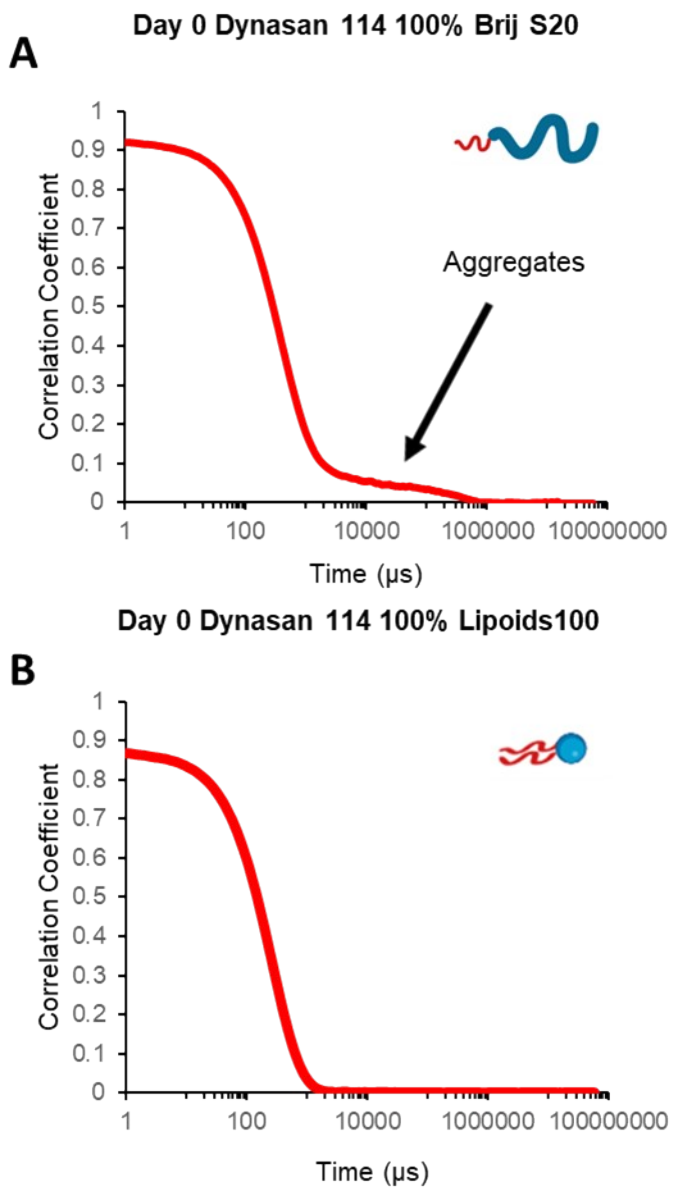

Fig. S2- Data collected by DLS on day 0 displaying size correlation curves of A) Dynasan 114 stabilised by pegylated lipid surfactant (brij 78) showing signs of aggregation or sedimentation due to presence of hump on curve, and B) Dynasan 114 stabilised by unpegylated lipid surfactant (Lipoid S100).

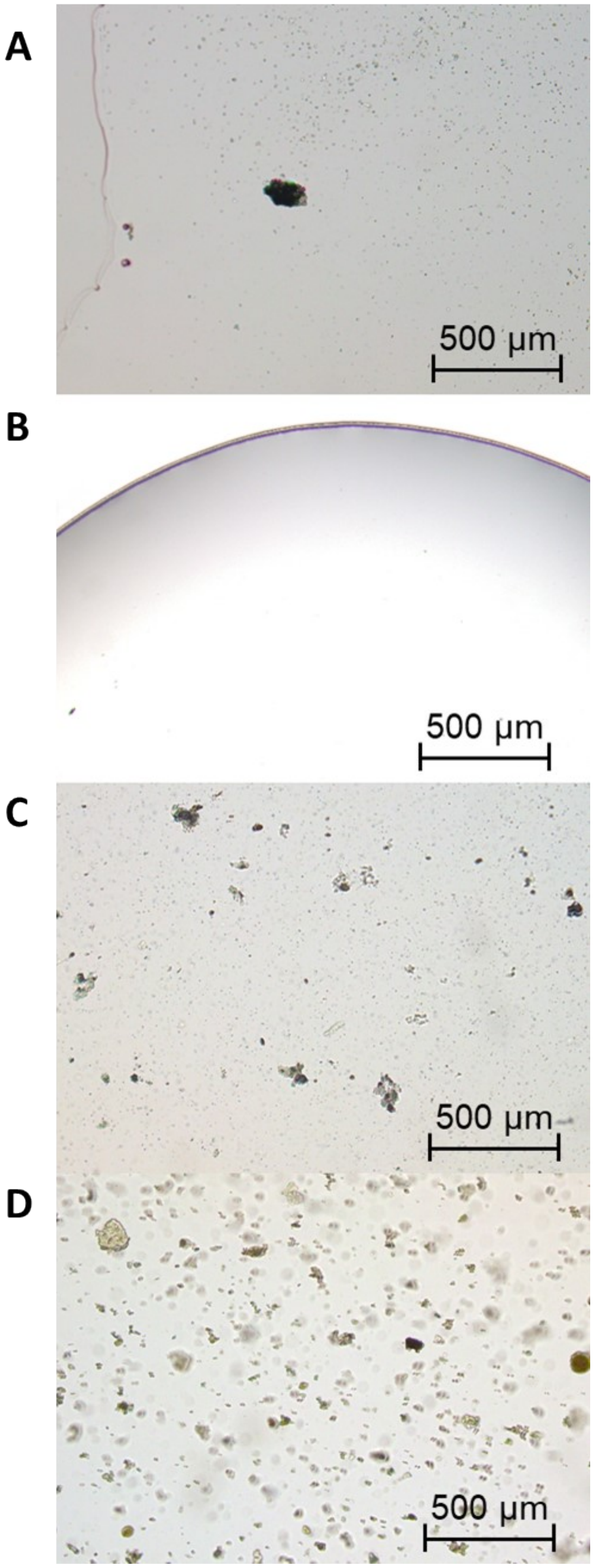

Fig. S3- Data collected by optical microscopy on day 2 comparing samples prepared by both Brij78 and Lipoid S100 for trimyristin and trimyristin. A) Brij78/trimyristin, B) Lipoid S100/trimyristin, C) Brij78/tristearin, D) Lipoid S100/tristearin.

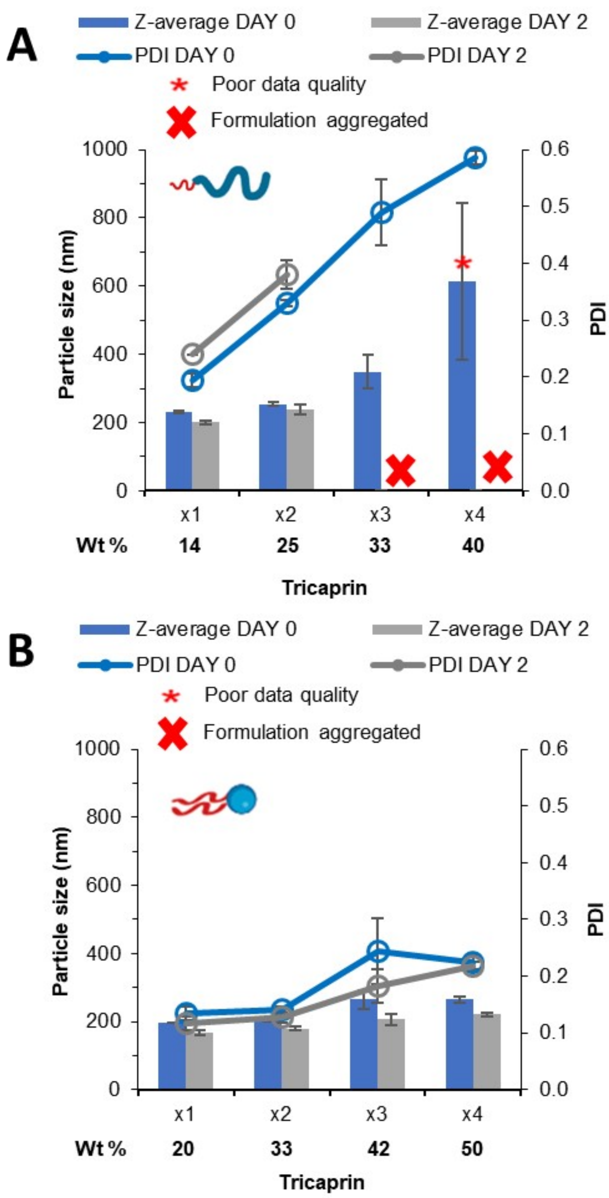

Fig.S4- Data collected measuring both particle size and size distribution by DLS. Formulations of Tricaprin at increasing wt % stabilised by A) Pegylated lipid surfactant Brij S20 B) Unpegylated lipid surfactant Lipoid S100. Both surfactants were used at equal mol %.

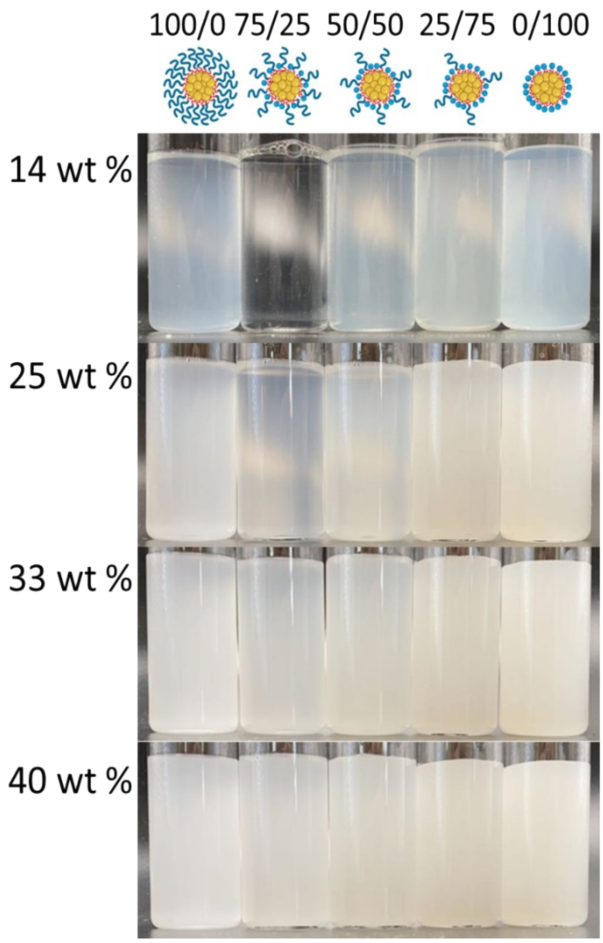

Fig. S5- Displays visual changes in the appearance of formulations. From left to right displays variation between surfactant composition within the formulation, while from top to bottom displays variation between wt % of tricaprin in formulation. Overall images display how surfactant composition and wt % may influence formulation turbidity as a result of varying degrees of light scattering caused by means of particle size and concentration of particles.

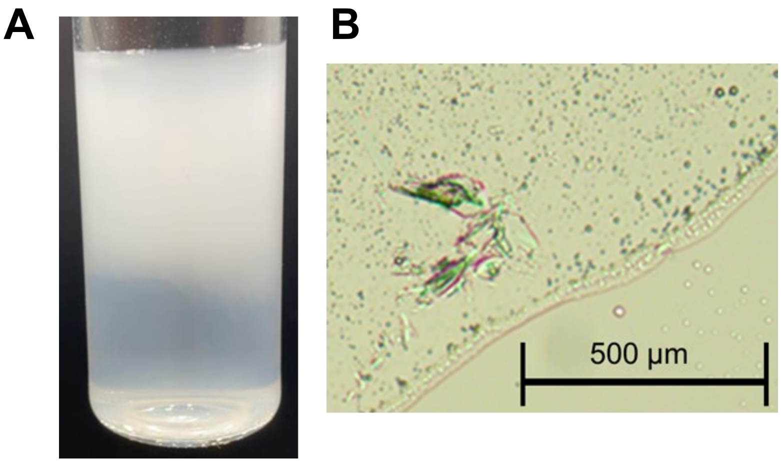

Fig. S6- Displays A) Photo of 100 % brij 78 33 wt % tricaprin formulation on day 6 which has experienced phase separation. B) Optical microscopy image of the top phase indicating the presence of aggregates

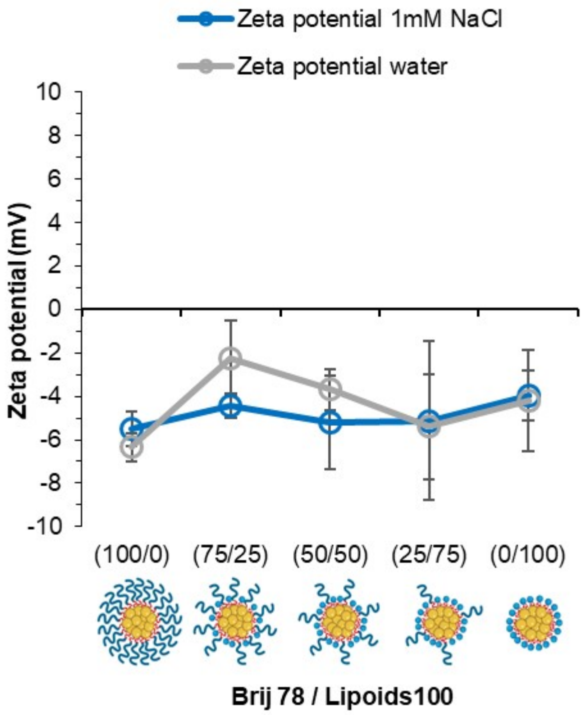

Fig. S7- Data collected measuring zeta potential on day 2 of formulation of tricaprin formulations measured at 14 wt% for each surfactant composition of Brij S20 and Lipoid S100

A

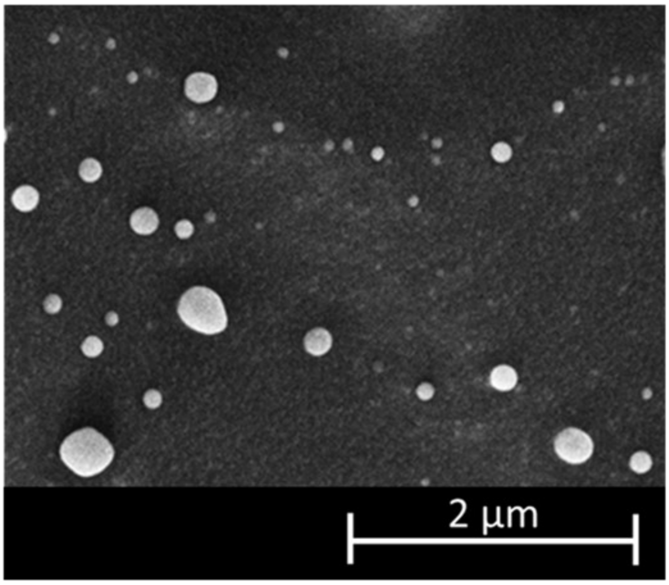

Fig. S8- Cryo-SEM image of 100 % tricaprin nanoparticles at 14 wt % stabilised by 50 % Brij S20 50 % Lipoid S100.

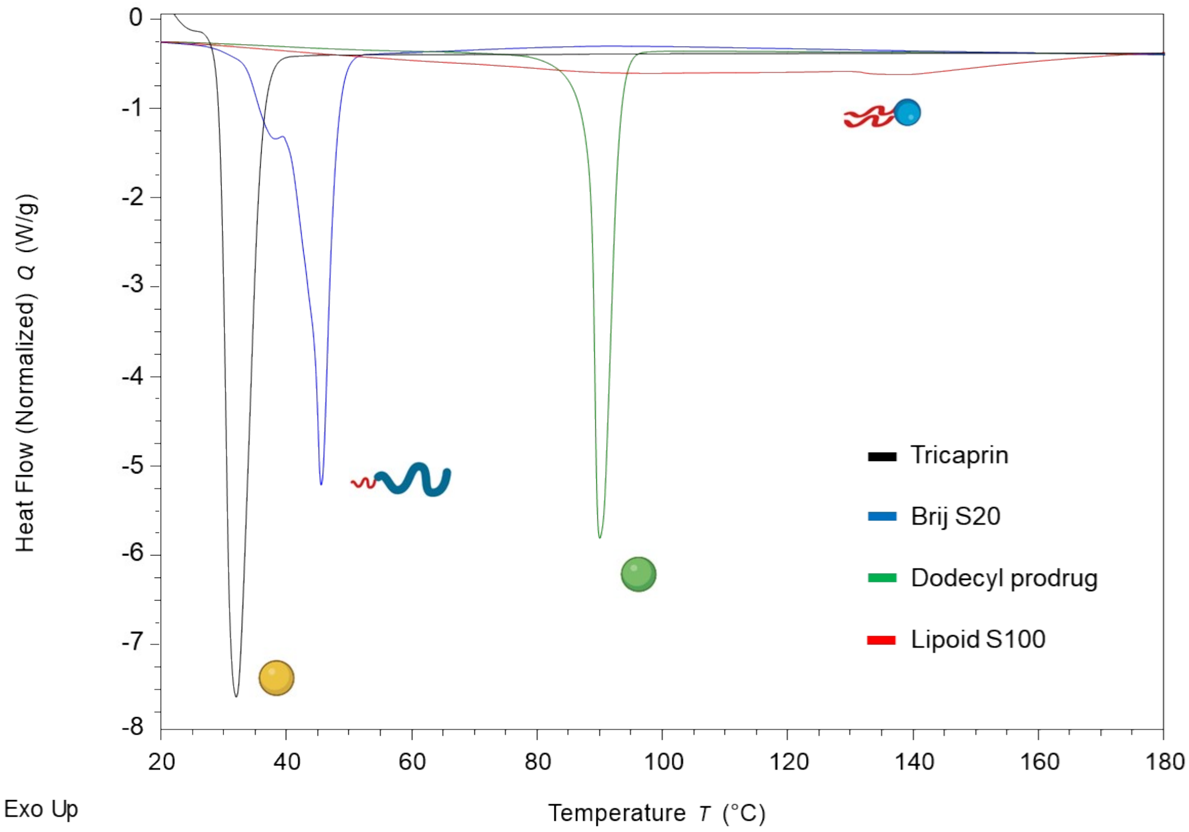

Fig. S9- Displays the thermal properties of each of the materials in bulk.

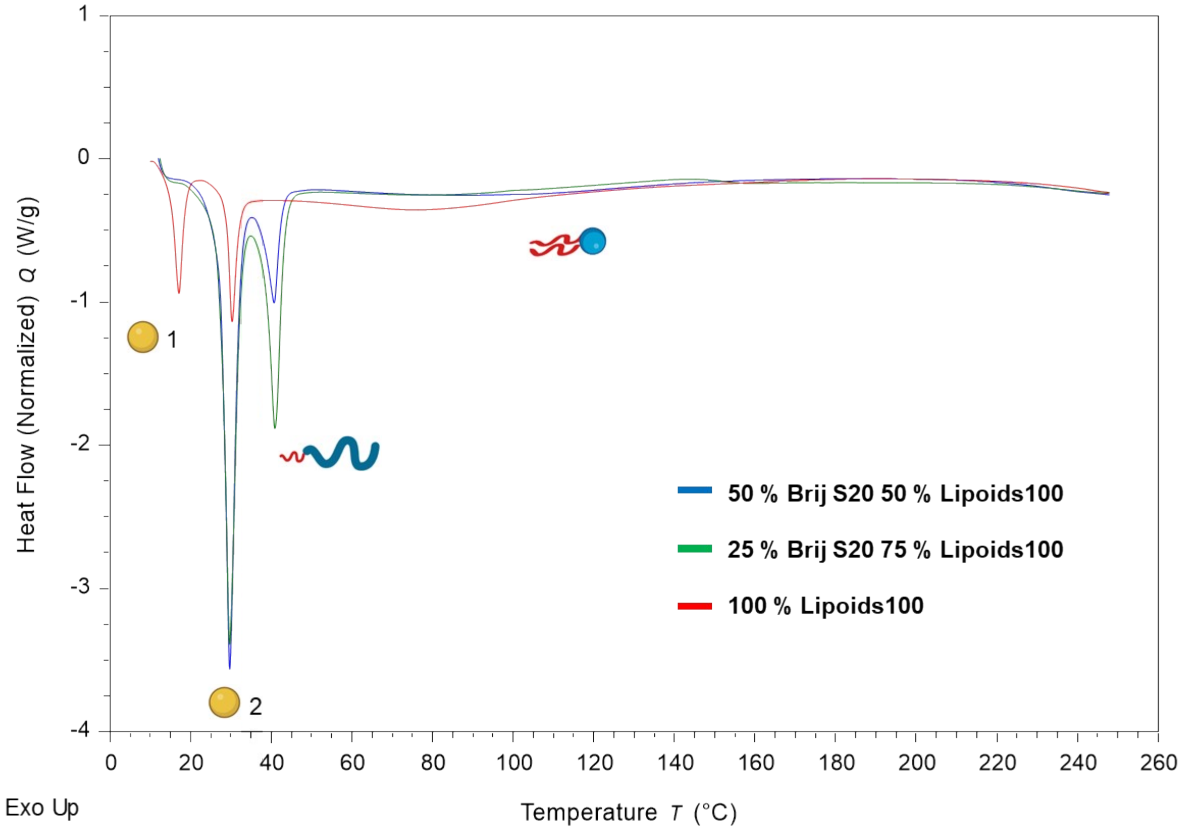

Fig. S10- Displays an overlay of thermograms for each surfactant blend at 40 wt % tricaprin

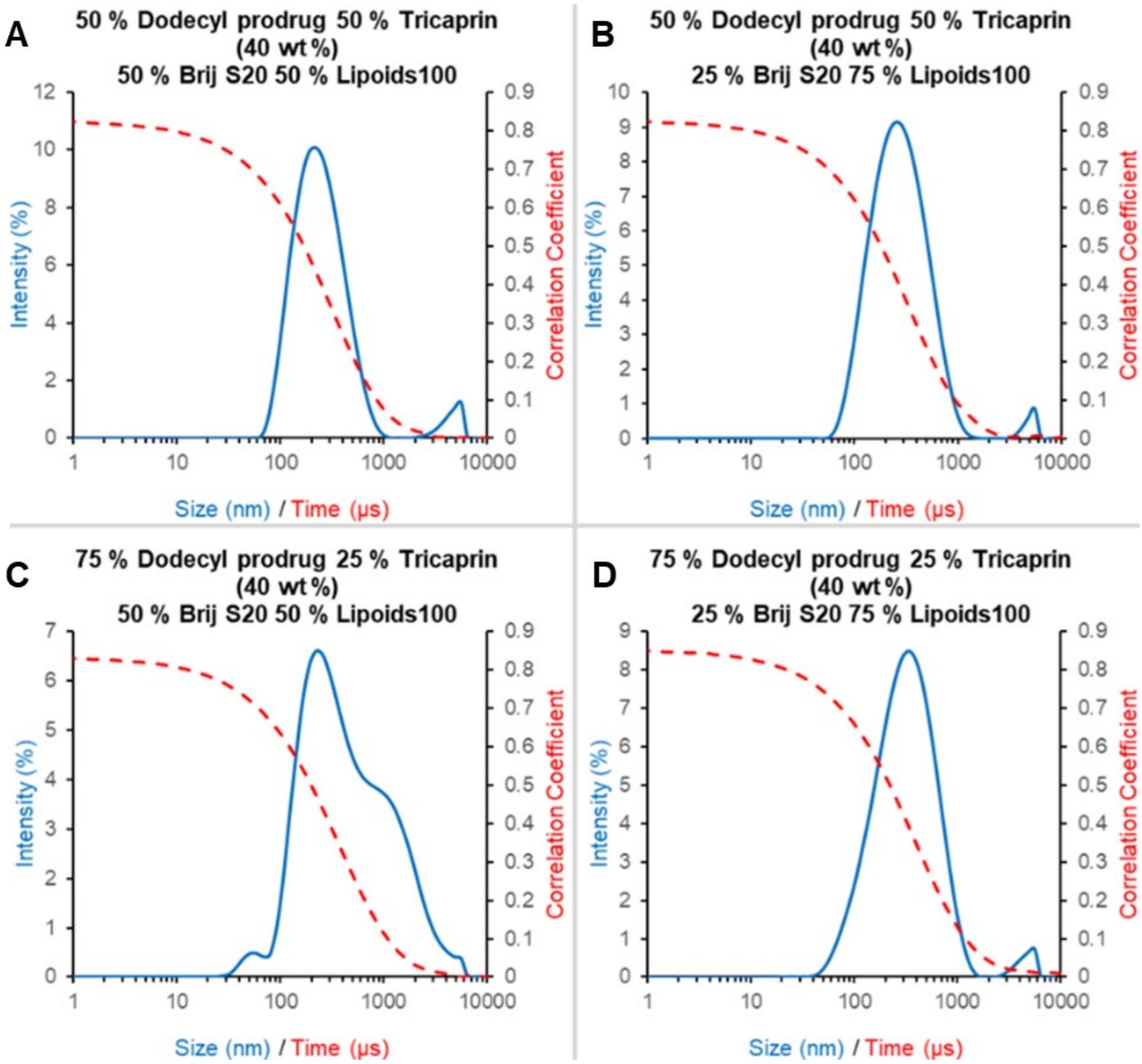

Fig. S11- Size distribution graph and correlation coefficient curves corresponding to each of the formulations in Fig 10. Size distribution graphs were created by taking an average of the value for each of the triplicate measurements. Dodecyl prodrug was formulated at 50 % Dodecyl prodrug loading for A and B, at a surfactant blend of 50 % pegylated lipid 50 % unpegylated lipid surfactant (A); and 25 % pegylated lipid 75 % unpegylated lipid surfactant (B). At 75 % Dodecyl prodrug loading for C and D at a surfactant blend of 50 % pegylated lipid 50 % unpegylated lipid surfactant (C); and 25 % pegylated lipid 75 % unpegylated lipid surfactant (D).

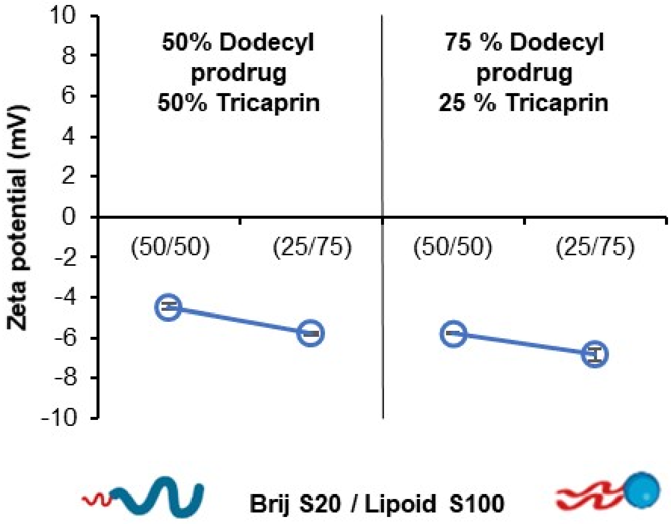

Fig. S12- Data collected measuring zeta potential on day 2 of formulation of dodecyl prodrug/ tricaprin blend formulations measured at 40 wt% for each surfactant composition of Brij S20 and Lipoid S100

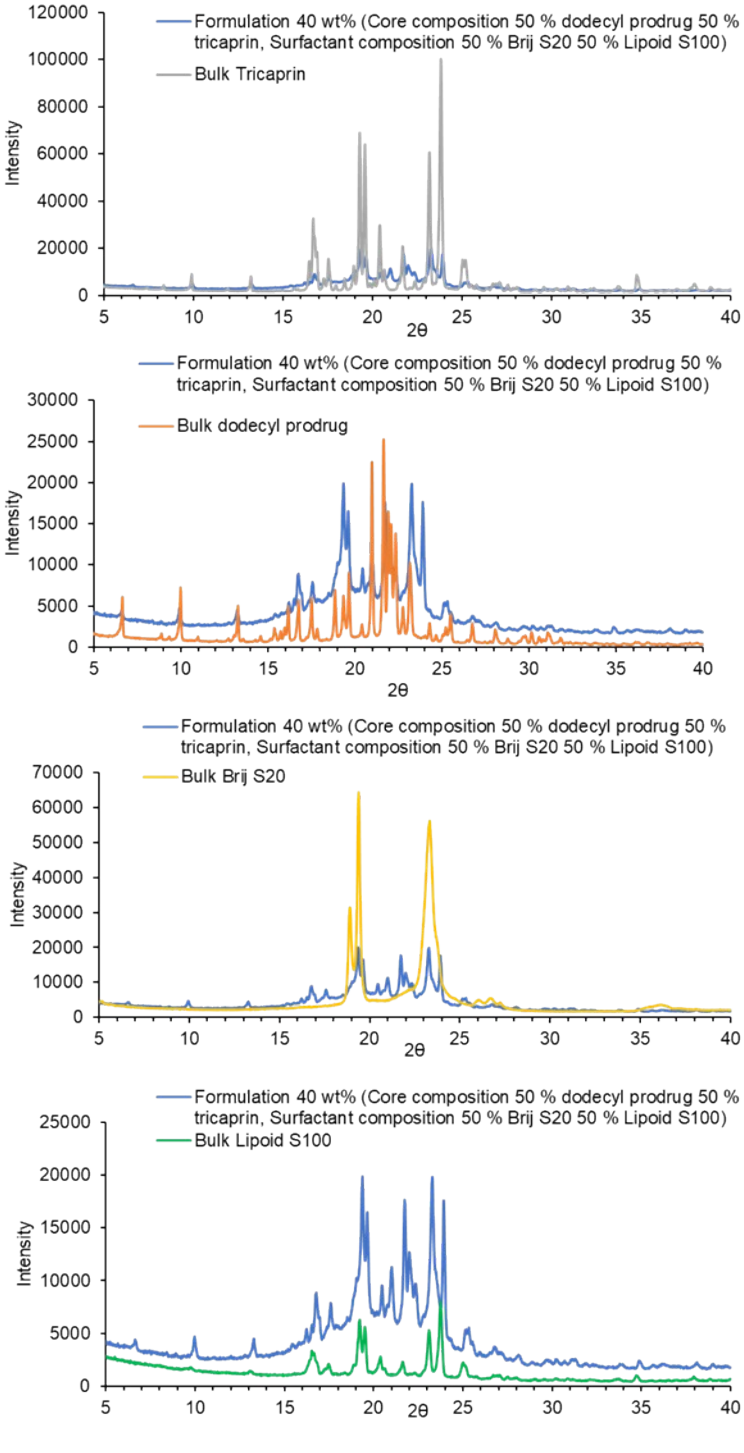

Fig. S13- Displays an overlay of PXRD traces of the formulation shown in Fig.10 with the individual bulk components (grey, tricaprin; orange, dodecyl prodrug; yellow, Brij S20 and green, Lipoid S100)
